# Cross-cultural adaptation and measurement properties of generic and cancer-related patient-reported outcome measures (PROMs) for use with cancer patients in Brazil: a systematic review

**DOI:** 10.1007/s11136-017-1703-5

**Published:** 2017-09-08

**Authors:** Carlos Augusto Albach, Richard Wagland, Katherine J. Hunt

**Affiliations:** 1 Faculty of Health Sciences, University of Southampton, University Road, Southampton, Hampshire SO17 1BJ UK; 2 Faculty of Health Sciences, University of Southampton, Highfield, Southampton, Hampshire SO17 1BU UK

**Keywords:** Patient-reported outcome measures, Brazil, Portuguese, Cancer, Cross-cultural adaptation, Measurement properties

## Abstract

**Purpose:**

This systematic review (1) identifies the current generic and cancer-related patient-reported outcome measures (PROMs) that have been cross-culturally adapted to Brazilian Portuguese and applied to cancer patients and (2) critically evaluates their cross-cultural adaptation (CCA) and measurement properties.

**Methods:**

Seven databases were searched for articles regarding the translation and evaluation of measurement properties of generic and cancer-related PROMs cross-culturally adapted to Brazilian Portuguese that are applied in adult (≥18 years old) cancer patients. The methodological quality of included studies was assessed using the COSMIN checklist.

**Results:**

The bibliographic search retrieved 1674 hits, of which seven studies analysing eight instruments were included in this review. Data on the interpretability of scores were poorly reported. Overall, the quality of the CCA process was inconsistent throughout the studies. None of the included studies performed a cross-cultural validation. The evidence concerning the quality of measurement properties is limited by poor or fair methodological quality. Moreover, limited information regarding measurement properties was provided within the included papers.

**Conclusions:**

This review aids the selection process of Brazilian Portuguese PROMs for use in cancer patients. After acknowledging the methodological caveats and strengths of each tool, our opinion is that for quality of life and symptoms assessment the adapted FACT-G version and the ESAS could be recommended, respectively. Future research should rely on the already accepted standards of CCA and validation studies.

**Electronic supplementary material:**

The online version of this article (doi:10.1007/s11136-017-1703-5) contains supplementary material, which is available to authorized users.

## Introduction

Patient-reported outcome measures (PROMs) are a method for ascertaining patients’ views of their symptoms, functional status and health-related quality of life (HRQoL), and are useful in research to identify the subjective impact of medical or psychosocial interventions upon patients. Recently in the US and the UK, PROMs have been used to assess and compare health outcomes achieved by different healthcare providers [1]. Currently, health-related PROMs (HR-PROMs) are widely used and have a well-established role in clinical practice, research, audit and health policy. Measuring patients’ subjective health status and quality of life (QoL) has also become increasingly important as life expectancy has risen in developed and underdeveloped countries, increasing the prevalence of chronic disease. Today, cardiovascular disease and cancer are the most common causes of death in Brazil [2], with 596,000 new cancer cases expected in 2016 [3]. Moreover, cancer patients frequently have long and complex disease trajectories making measurement of patient-reported and clinical outcomes equally important.

PROM is an umbrella term for the vast range of health-related questionnaires that are completed by patients. Generic PROMs are tailored to assess relevant health domains to all individuals, thus allowing comparisons of health status between different populations and disease groups [4]. Similarly, cancer-related PROMs can be applied across distinct tumour types. Whenever possible, the use of an existing validated PROM is preferred rather than developing a new scale [5]. However, most instruments have been developed and validated in English-speaking populations [6, 7], so many have subsequently been adapted for other languages and cultures.

Cross-cultural adaptation (CCA) and validation (CCV) of PROMs is a contentious issue due to various possible methodological approaches [8]. Epstein et al. differentiate the term translation (the production of a document from a source to a target language) from CCA (a process that ensures equivalence in meaning) [8]. CCV could be described as ‘the degree to which the performance of the items on a translated or culturally adapted PROM instrument is an adequate reflection of the original version of the PROM’ [9]. A well-conducted CCA is critical to ensure a good methodological quality of CCV. There has been a growing interest in cross-culturally adapted and validated HR-PROMs for use in multinational trials recruitment and external validation of effectiveness studies [10].

Portuguese ranks sixth as the most international language, with 206 million native Portuguese speakers worldwide [11], the majority of them in Brazil. In 2007, 1.2 million Brazilians lived in the United States and an additional 2.8 million people were living outside America [12]. There are an increasing number of PROMs available in Brazilian Portuguese, providing a wide choice in the selection of suitable instruments. Whilst systematic reviews of HR-PROMS are recognised as valuable to aid in the selection of robust instruments [13], there has only been one review assessing CCA of Brazilian Portuguese PROMs focused solely on shoulder disability. The authors highlighted a lack of robust critical evaluation of instruments in Brazil [14]. The objectives of this systematic review, therefore, are to identify generic and cancer-related PROMs that have been cross-culturally adapted to Brazilian Portuguese and assessed for use with cancer patients, and to critically appraise the quality of CCA and measurement properties of translated versions with a view at informing potential users both in clinical research and in practice settings.

## Methods

### Search strategy

The search for articles was performed using an adapted strategy that involved the combination of free text search terms, medical subject headings (MeSH) for terms in English, health sciences descriptors for terms in Portuguese (including patient-reported, quality of life, cancer, cross-cultural, language, Brazil and Portuguese) and the Boolean operators ‘OR’, ‘AND’ and ‘NOT’ [15]. The following databases were searched: MEDLINE (OVID, 1946 onwards), EMBASE (OVID, 1947 onwards), PsycINFO (EBSCO, 1800 s onwards), CINAHL Plus (EBSCO, 1937 onwards) and Scopus (1960 onwards). For Latin America databases SciELO (Web of Science) and LILACS, the same text words were used, albeit in Portuguese. All databases were set up to run a new search every week until the 1st of June 2016. The MEDLINE (OVID) search strategy is included in Online Resource 1. The strategy was adapted to the syntax of other databases and is available upon request. The reference list of included studies was manually scanned for additional studies.

Results from the literature search were uploaded to EndNote software v.7.5 (Thomson Reuters^®^) by the lead reviewer (CA) and were assessed for eligibility by the second reviewer (RW), differences being resolved in discussion with a third reviewer (KJH). Duplicated hits were excluded, and the remaining records were screened by title and abstract. All studies deemed to be eligible had their full text obtained. The reasons for exclusion were recorded. A maximum of two e-mail attempts to contact authors were made to collect any missing information.

### Selection criteria

A study was considered eligible if it met the following inclusion criteria: original article (e.g. not a conference abstract, review or editorial), published in English or Portuguese, aimed primarily at evaluating the measurement properties of a generic or cancer-related PROM adapted to Brazilian Portuguese for use with adult (≥18 years old) cancer patients in clinical trials or in oncology care delivery. There were no restrictions concerning primary site or stage of disease, treatment, gender or other clinical or epidemiological characteristics. Publications describing solely the CCA process were excluded, as well as studies reporting on disease-specific PROMs other than cancer and domain-specific questionnaires.

### Data collection and critical appraisal of eligible studies

The following data of the included studies were extracted: year of publication, inclusion and exclusion criteria, sample size, PROM used, full-text availability of the tool, number of items of the instrument and the reported time to complete the questionnaire. The epidemiological and clinical characteristics of the subjects included in each study (age, gender, cancer stage, disease status, treatment received and setting) were extracted. Information relevant to the interpretability of the instrument (percentage of missing items, distribution of scores and percentage of the respondents who had the highest and lowest possible scores) was collected.

**Fig. 1:**
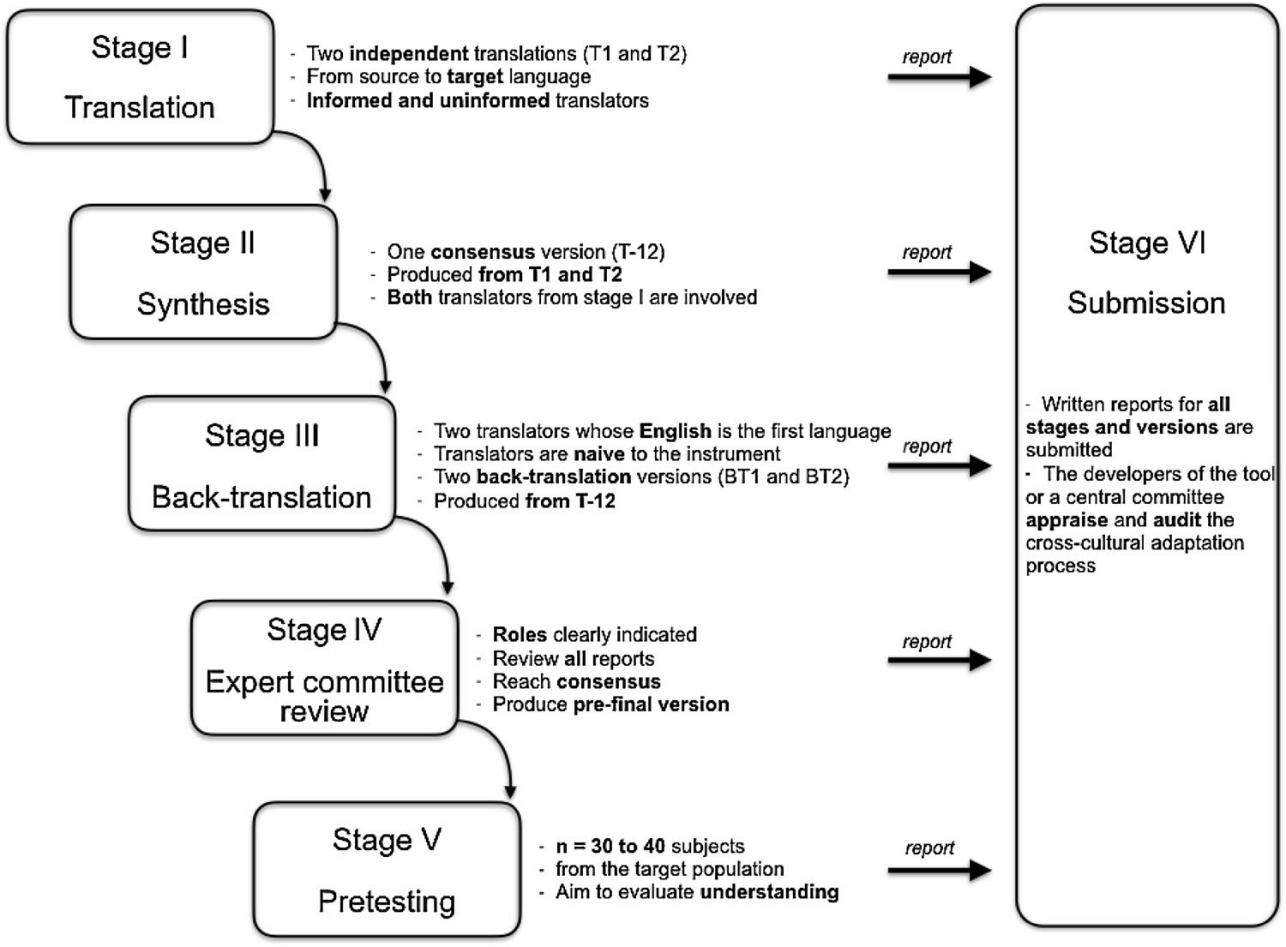
Recommended stages for cross-cultural adaptation (adapted from Beaton et al. [16])

The CCA quality was independently assessed by both CA and RW, based on the stages described by Beaton et al. [16] (Fig. 1). Table 1 shows the rating method for quality assessment of CCA [7]. The original study that described the full CCA process was retrieved and evaluated when the CCA and the measurement properties of the same PROM involved separate publications.

**Table 1:** Quality criteria of the cross-cultural adaptation process (adapted from Oliveira et al. [7])

Stage	Rating^a^	Quality criteria
I: Forward translation	+	Translations conducted by two or more independent translators
	?	Doubtful translation process (e.g. translators’ background or awareness status about the tool are different from the recommended, translation conducted by one translator)
	−	Translation conducted by two non-independent translators
	0	No information on the forward translation process
II: Synthesis	+	Synthesis conducted by the same two or more translators from stage I
	?	Doubtful synthesis process (e.g. different translators or professionals from stage I)
	0	No information on the synthesis process
III: Back-translation	+	Back-translation made by two or more independent translators for whom English is the first language and who are naive to the instrument
	?	Doubtful back-translation process (e.g. English is not the translators’ first language or they are aware of the instrument, back-translation conducted by one translator only)
	−	Back-translation made by two non-independent translators
	0	No information on back-translation process
IV: Expert committee review	+	An expert committee is reported and participants’ roles clearly indicated. The committee reviews all documents
	?	Doubtful expert committee review (e.g. there is no mention of participants’ roles)
	−	The committee reviews only one or some documents
	0	No information on expert committee
V: Pretesting	+	Pretest was conducted in 30 or more subjects from the target population
	?	Doubtful design (e.g. there is no mention of the number of subjects tested, target population not described)
	−	Pretest was conducted in less than 30 subjects
	0	No information on the pretest
VI: Submission	+	All reports and forms were submitted to the developer of the instrument or central committee for appraisal
	?	Doubtful submission process (e.g. the reports and forms were received by others instead of the developer of the instrument or central committee)
	0	No information on submission process

^a^ + Positive rating; ? indeterminate rating; − negative rating; *0* no information available

The measurement properties described in all eligible studies were first identified using the taxonomy recommended by Mokkink et al. [9]. Methodological quality of included studies was assessed using the COnsensus-based Standards for the selection of health Measurement INstruments (COSMIN) scale [17, 18]. The scale has previously been used in validation studies [19] and systematic reviews of PROMs [20, 21]. The checklist contains twelve boxes. Boxes A to I contain standards for studies on measurement properties. Four different answer options for each item of boxes A to I are possible (excellent, good, fair and poor), representing the methodological quality of that specific item. Finally, a methodological quality score per box is achieved by selecting the lowest rating of any item in a box. The full-text score system is available at www.cosmin.nl.

Finally, measurement properties from each adapted PROM were independently graded by CA and RW as positive, indeterminate or negative based on pre-specified criteria [22, 23] (Table 2), again with disagreements resolved by KJH.

**Table 2:** Quality criteria for measurement properties of health-related questionnaires [23] (based on Terwee et al. [22])

Property	Rating^a^	Quality criteria
Reliability		
Internal consistency	+	(Sub)scale unidimensional AND Cronbach’s alpha(s) ≥0.70
	?	Dimensionality not known OR Cronbach’s alpha not determined
	−	(Sub)scale not unidimensional OR Cronbach’s alpha(s) <0.70
Measurement error	+	MIC > SDC OR MIC outside the LOA
	?	MIC not defined
	−	MIC ≤ SDC OR MIC equals or inside LOA
Reliability	+	ICC/weighted Kappa ≥0.70 OR Pearson’s *r* ≥ 0.80
	?	Neither ICC/weighted Kappa nor Pearson’s r determined
	−	ICC/weighted Kappa < 0.70 OR Pearson’s *r* < 0.80
Validity		
Content validity	+	The target population considers all items in the questionnaire to be relevant AND considers the questionnaire to be complete
	?	No target population involvement
	−	The target population considers items in the questionnaire to be irrelevant OR considers the questionnaire to be incomplete
Construct validity		
Cross-cultural validity	+	Original factor structure confirmed OR no important DIF
	?	Confirmation of original factor structure AND DIF not mentioned
	−	Original factor structure not confirmed OR important DIF
Structural validity	+	Factors should explain at least 50% of the variance
	?	Explained variance not mentioned
	−	Factors explain <50% of the variance
Hypothesis testing	+	(Correlation with an instrument measuring the same construct ≥0.50 OR at least 75% of the results are in accordance with the hypotheses) AND correlation with related constructs is higher than with unrelated constructs
	?	Solely correlations determined with unrelated constructs
	−	Correlation with an instrument measuring the same construct <0.50 OR < 75% of the results are in accordance with the hypotheses OR correlation with related constructs is lower than with unrelated constructs
Responsiveness		
Responsiveness	+	(Correlation with an instrument measuring the same construct ≥0.50 OR at least 75% of the results are in accordance with the hypotheses OR AUC ≥0.70) AND correlation with related constructs is higher than with unrelated constructs
	?	Solely correlations determined with unrelated constructs
	−	Correlation with an instrument measuring the same construct <0.50 OR < 75% of the results are in accordance with the hypotheses OR AUC < 0.70 OR correlation with related constructs is lower than with unrelated constructs

*MIC* minimal important change, *SDC* smallest detectable change, *LOA* limits of agreement, *ICC* intraclass correlation coefficient, *DIF* differential item functioning, *AUC* area under the curve

^a^+ positive rating; ? indeterminate rating; − negative rating

## Results

**Fig. 2:**
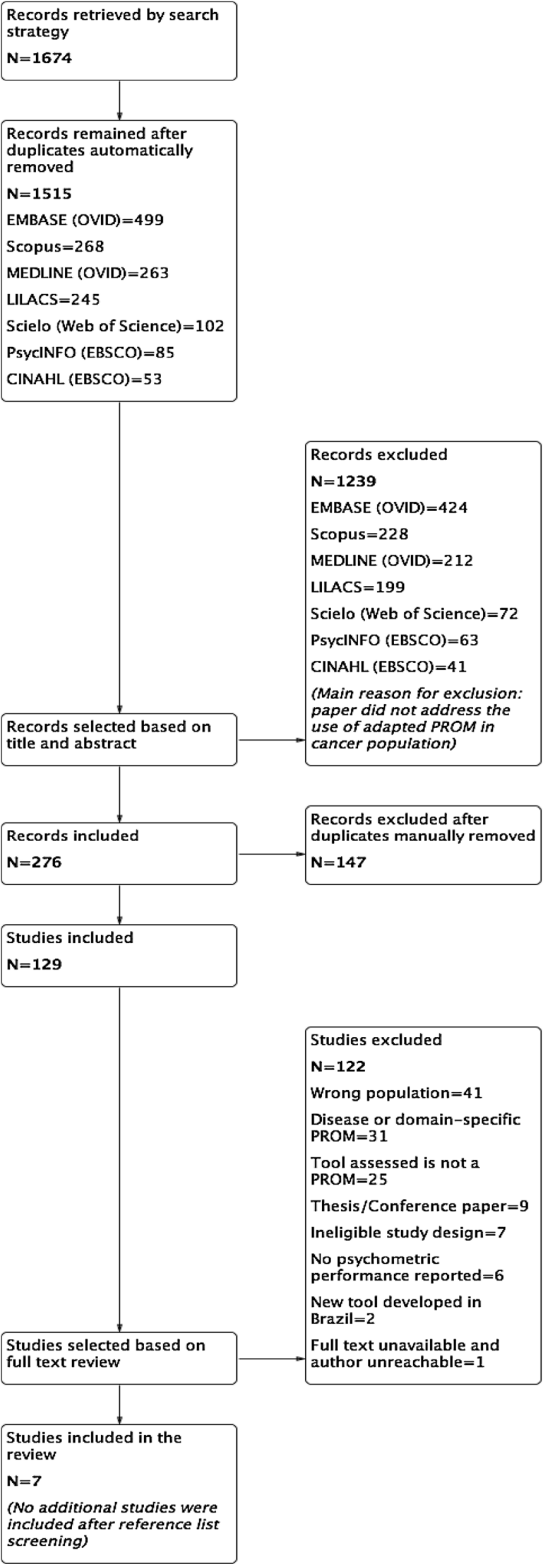
Flowchart of database search strategy and selection of studies

The database search resulted in 1674 records (Fig. 2). The first, automatic round of duplicates screening removed 159 records. Hence, 1515 records remained to be analysed by title and abstract, of which 1239 records were excluded mainly because the paper did not describe the use of a cross-culturally adapted PROM in Brazilian cancer patients or the study was not concerned with PROMs at all. Then, 276 records were submitted to a manual analysis of duplicated hits, resulting in 147 redundant records. Finally, 129 studies had their full text analysed against the inclusion and exclusion criteria: 122 studies were excluded and seven studies met the inclusion criteria following full-text review. Online Resource 2 details the excluded studies following full-text assessment. An Excel spreadsheet file with additional data extracted from the excluded records is available upon request.

In total, eight HR-PROMs were identified: four health-related QoL tools (two European Organization for Research and Treatment of Cancer QoL questionnaires—EORTC QLQ-C30 [24] and EORTC QLQ-C15-PAL [25], Functional Assessment of Cancer Therapy-general or FACT-G [26] and the World Health Organization QoL questionnaire short version or WHOQOL-bref [27]); one symptom assessment tool (Edmonton Symptom Assessment System or ESAS [28]); one symptom and severity assessment tool (MD Anderson Symptom Inventory-core or MDASI [29]); one health status tool (Medical Outcomes Study 36-Item Short-Form Health Survey or SF-36 [27]) and one functional screening tool (Vulnerable Elders Survey-13 or VES-13 [30]).

Table 3 shows the general characteristics of studies. The full text of the adapted instrument was provided by only one study [28]. The average time to complete the questionnaire was described only for the ESAS [28].

**Table 3:** Characteristics of included studies

Instrument	Instrument full-text availability	Instrument total number of items	Mean time to complete the instrument	Actual sample size (analysed data)
FACT-G [26]	Yes^a^	27	NR	975
MDASI [29]	Yes^b^	19	NR	268
VES-13 [30]	Yes^c^	13	NR	272 108^d^
EORTC QLQ-C-15-PAL [25]	No	15	NR	104
SF-36 [27]	No	36	NR	106 99^e^ 94^f^
WHOQOL-bref [27]	No	24	NR	106 99^e^ 94^f^
EORTC QLQ-C30 [24]	No	30	NR	986
ESAS [28]	Yes	10	2.24 min	249 84^g^ 80^h^

*NR* not reported

^a^ As per request to Functional Assessment of Chronic Illness Therapy (FACIT) Group

^b^ In a separate publication [32]

^c^ In a separate publication [33]

^d^ Sample size for the reliability measurement (assessed 7 to 15 days after baseline)

^e^ Sample size for the reliability measurement (assessed 48 h after baseline)

^f^ Sample size for the responsiveness measurement (assessed 30 days after baseline)

^g^ Sample size for the reliability measurement (assessed 4 to 96 h after baseline)

^h^ Sample size for the responsiveness measurement (assessed 21±7 days after baseline)

Table 4 presents data relevant to the interpretability of the studies. Adequate description about how missing items were handled was found in one study [26]. Campos et al. reported the missing data to FACT-G items (i.e. the non-response rate) ranging from 0.1 to 0.6%, except for the item related to ‘satisfaction with the sex life’. This item had a non-response rate of 45% and was excluded from the final analysis after consent from the developers of the original tool. Moreover, the authors opted to exclude individuals who did not answer one or more of the other PROM items [26]. The distribution of scores was infrequently reported across studies.

**Table 4:** Interpretability of included studies

Instrument	Missing scores	Description of how missing items were handled	Distribution of total scores	Respondents with the lowest possible total score	Respondents with the highest possible total score
FACT-G [26]	0.1 to 0.6% of non-response rate per item^a^	Yes	NR	NR	NR
MDASI [29]	NR	NR	NR	NR	NR
VES-13 [30]	NR	NR	NR	NR	NR
EORTC QLQ-C-15-PAL [25]	NR	NR	NR	NR	NR
SF-36 [27]	NR	NR	Yes	Yes	Yes
WHOQOL-bref [27]	NR	NR	Yes	Yes	Yes
EORTC QLQ-C30 [24]	0.084% of missing items per questionnaire	No	NR	NR	NR
ESAS [28]	One non-response item of out 249 questionnaires	No	Yes	NR	NR

*NR* not reported

^a^ Except for item 14 (‘satisfaction with sexual life’), which had 45% of non-response rate and was removed from the analysis

Factors influencing the generalisability of the tools are presented in Table 5. All PROMs have been used with patients attending outpatient departments, while FACT-G and ESAS have also been used with inpatients [26, 28]. The EORTC QLQ-C-15 PAL was applied only in patients with advanced cancer. Limited participant description hinders the generalisability of VES-13 and EORTC QLQ-C-30 [24, 30].

**Table 5:** Generalisability of included studies^a^

Instrument	Age (years, mean ± SD) Gender (Male %)	Setting	Cancer stage (TNM)	Disease status	Treatment
FACT-G [26]	53.3 ± 13.0 38.5%	Outpatient Inpatient	I–IV	Active	CT/HT/RT/IMT/ Any combination of above
MDASI [29]	61.5 ± 14.9 35.8%	Outpatient	III and IV: 51.8%^b^	Active	CT/RT/surgery
VES-13 [30]	71.6 ± 0.4 61.6%	Outpatient	NR	NR	NR
EORTC QLQ-C-15-PAL [25]	57.5 ± NR 45.1%	Outpatient	IV	Active	CT/surgery were cited as the ‘most common’ used
SF-36 and WHOQOL-bref [27]^c^	49.2 ± 9.6 NA	Outpatient	IV: 24.5%^b^	NR	CT/HT/RT/surgery
EORTC QLQ-C30 [24]	57 ± 13 41.7%	NR	NR	NR	CT/exclusive palliative care
ESAS [28]	55.1 ± 12.6 36.5%	Outpatient Inpatient	“Advanced” and stage IV: 87.6%	Active	CT/RT/exclusive palliative care

*CT* chemotherapy, *HT* hormonal therapy, *IMT* immunotherapy, *NA* not applicable, *NR* not reported, *RT* radiotherapy

^a^ All authors used the convenience sample method, except for Paiva et al. [24] and Nunes [25] (both not reported)

^b^ The relative percentages of other stages were not reported

^c^ Female breast cancer population

Table 6 shows the CCA quality of the HR-PROMs identified. No studies achieved positive ratings in more than three of the six criteria, with back-translation being the strongest single criterion amongst studies. Although the expert committee review stage was positive for two instruments (EORTC QLQ-C30 and ESAS), only the ESAS adaptation study had the committee composition clearly described, consisting of a medical oncologist, a registered nurse, a dentist, an occupational therapist and a psychologist.

**Table 6:** Cross-cultural adaptation quality of generic and cancer-related Brazilian Portuguese PROMs applied for use with cancer patients

Instrument	Pilot test sample (*N*)	Forward translation	Synthesis	Back-translation	Expert committee review	Pretesting	Submission
FACT-G^a^ [34]	32	+	+	?	?	+	?
ESAS [28]	24	+	?	+	+	–	?
EORTC QLQ-C30^a^ [36]	NR^b^	?	?	+	+	–	+
SF-36^a^ [35]	20	?	+	+	?	–	0
VES-13^a^ [33]	33	?	0	?	?	+	0
MDASI^a^ [32]	NR	?	?	?	?	0	?
EORTC QLQ-C-15-PAL [25]	NR	?	0	?	–	?	0
WHOQOL-bref [27]	Unavailable data						

*NR* not reported

^a^ Cross-cultural adaptation and measurement properties assessment of the instrument involved two separate publications

^b^ Sample size was not reported. However, the EORTC quality of life translation manual recommends 10 to 15 subjects as a general rule [36]

Both the methodological grade and the quality of measurement properties are presented together in Table 7. Cross-cultural validity (i.e. the equivalence of scores between the original and target populations, usually assessed by multiple group factor analysis or item response theory techniques [31]) was not reported in any study. Evidence for excellent or good methodological quality with a positive result for any measurement property was uncommon.

**Table 7:** Methodological grade and quality of the measurement properties of cancer-related and generic Brazilian Portuguese PROMs for use with cancer patients

	Instrument	Methodological grade (excellent/fair/poor)^a^ and quality of measurement property (+/−/?)^b^						
		Validity			Reliability			Responsiveness
		Content	Structural	Hypothesis testing	Internal consistency	Reliability	Measurement error	
FACT-G	Methodological quality	Excellent	Excellent	Fair	Excellent			
[26]	Measurement quality	+	?	–	+			
MDASI	Methodological quality		Fair		Fair			
[29]	Measurement quality		–		–			
VES-13	Methodological quality	Excellent	Fair	Poor		Fair		
[30]	Measurement quality	+	+	–		+		
EORTC QLQ-C-15-PAL	Methodological quality		Fair	Fair	Fair			
[25]	Measurement quality		?	–	–			
SF-36	Methodological quality			Fair	Poor	Fair	Fair	Poor
[27]	Measurement quality			–	?	–	?	–
WHOQOL-bref	Methodological quality			Fair	Poor	Fair	Fair	Poor
[27]	Measurement quality			+	?	+	?	+
EORTC QLQ-C30	Methodological quality			Good	Poor			
[24]	Measurement quality			+	?			
ESAS	Methodological quality			Good	Good	Poor		Poor
[28]	Measurement quality			+	?	+		?^c^

^a^ Assessed using the COSMIN scale (available at www.cosmin.nl)

^b^ + Positive rating; ? indeterminate rating; − negative rating (Table 2)

^c^ Unknown due to distinct methodology used (Anchor-based method)

### ESAS [28]

The CCA process scored three positive and two doubtful marks. Pretesting was negative due to the pilot test sample size (*n* = 24).

Poor methodological quality scores for reliability (test–retest reliability was assessed by a second interview 4 to 96 h after the baseline) and responsiveness (authors provided a limited description of the measurement properties of the comparator instrument) were observed. Hypothesis testing and internal consistency were of good methodological quality.

The result for internal consistency of the tool was indeterminate (dimensionality not confirmed [37]). On the other hand, there was evidence for a positive result in hypothesis testing (*r* > 0.52 for the correlation with similar items).

### EORTC QLQ-C30 [24]

Back-translation and expert committee review stages of the adaptation process were positive, while pretesting was negative due to the pilot test sample size. Questionable quality was scored for forward translation and synthesis stages [36].

Good methodological quality was observed for hypothesis testing. The methodological quality of internal consistency analysis was considered poor (factor analysis was not performed and no reference to another study was provided).

Evidence for a positive result in hypothesis testing was reported (85.1% of the previously hypothesised correlations were confirmed).

### EORTC QLQ-C15 PAL [25]

The CCA methodology analysis revealed a negative score in the expert committee review. Three stages scored doubtful marks, and no information was provided about the synthesis and submission steps.

The lack of description of missing scores hindered the methodological quality for structural validity and internal consistency. A fair hypothesis testing methodology was observed as the hypothesis about expected correlations between similar instruments was not specified a priori.

The result for structural validity (confirmatory factor analysis) was indeterminate due to the absence of factors explaining variance. There was evidence for a negative result in hypothesis testing (Spearman’s *r* = 0.30 for the correlation of EORTC QLQ-C15-PAL emotional functioning with a similar instrument) and internal consistency (Cronbach’s alpha = 0.58–0.86).

### FACT-G [26]

The CCA methodology has received three positive and three indeterminate marks [34].

Methodological quality of the measurement properties was rated as excellent for three out of four assessments. Few and vague hypotheses about the expected correlations of FACT-G with the EORTC QLQ-C30 were made a priori, hampering the methodological quality of hypothesis testing (construct validity).

There was evidence for a positive result in internal consistency (Cronbach’s alpha = 0.71–0.82). Low convergent validity was observed for the emotional and functional well-being domains. Confirmatory factor analysis provided evidence that the tool has a four-factor structure. However, the result of structural validity was indeterminate as the factors’ explained variance was not mentioned.

### MDASI [29]

The CCA methodology received doubtful rates in all but one step [32].

The methodological quality rate of both measurement properties assessed (structural validity and internal consistency) was fair due to the lack of description of missing scores.

Exploratory factor analysis showed that the MDASI Symptoms module had four factors, while the Interference module had a one-factor structure. The latter factor explained 49.9% of the variance, thus receiving a negative result (slightly below the set criteria of 50%). There was evidence for a negative result in internal consistency (Cronbach’s alpha <0.70 in all factors identified in the MDASI Symptoms module).

### SF-36 [27]

The CCA methodology received a range of different quality rates [35]. Synthesis and back-translation were positive, while pretesting was negative due to the pilot test sample size (20 subjects). Questionable quality was scored for forward translation and expert committee review. No information about the submission stage was provided.

The methodological quality rate of the measurement properties assessed was poor or fair. Internal consistency assessment methodology was poor because the authors did not provide evidence for unidimensionality of the (sub)scales. The lack of a priori hypothesis about changes in scores made the methodological quality of responsiveness analysis poor.

Indeterminate results were observed in measurement error (minimal important change not defined). Evidence for a negative result was observed in hypothesis testing (*r* < 0.50 between the scales of the SF-36 and similar scales of FACT-B + 4) and reliability (four dimensions with ICC_1,2_ < 0.7). Ceiling and floor effects have been detected.

### VES-13 [30]

The quality of the CCA process was questionable due to lack of information about synthesis and submission process and doubtful forward translation, back-translation and expert review committee stages [33].

A range of different rates was observed for the methodological quality of the validation study. Excellent quality was observed for content validity, while convergent and divergent validity methodology (hypothesis testing) was poor due to inadequate description of comparator instruments. Poorly described missing scores made the methodological quality of structural validity and reliability fair.

Principal component analysis indicated that the VES-13 has a three-factor structure, which explained 72.6% of sample variance (positive result). Reliability results were also rated positive (ICC for total VES-13 scores = 0.78).

### WHOQOL-bref [27]

The CCA methodology was not reported.

The methodological quality rate of measurement properties was identical to the SF-36 (both tools were evaluated in the same study).

There was evidence for a positive result in hypothesis testing (*r* = 0.61 to 0.69 with similar scales of FACT-B + 4) and reliability (ICC_1,2_ = 0.76 to 0.87). Indeterminate results were observed in measurement error (minimal important change not defined). There have been no ceiling or floor effects.

Overall, the quality of the CCA was variable across studies. The methodological quality of studies evaluating Brazilian Portuguese PROMs was mostly poor or fair, therefore limiting the evidence for the results of measurement properties. Content validity and measurement error were the least assessed measurement properties.

## Discussion

The number of HR-PROMs and their culturally and linguistically adapted versions has dramatically increased worldwide in recent years. This study aimed to synthesise the literature regarding CCA quality and measurement properties of cancer-related or generic, Brazilian Portuguese-adapted PROMs for use with cancer patients. The CCA quality throughout the eight identified instruments was inconsistent. Limited methodological quality was observed, thus compromising the measurement properties of the PROMs.

The majority of the identified instruments could be applied to adult cancer patients in Brazil. However, two instruments (SF-36 and WHOQOL-bref) were evaluated only in female breast cancer patients [27].

Interpretability refers to ‘what the scores (or changes in scores) on an instrument mean’ [38] and is key for the accurate use of PROMs. The interpretability of included studies was hampered as most of the authors have not reported relevant information. Bias in the validation of tools is therefore possible due to the limited description of missing scores. Underreporting of the highest and lowest scores for most instruments similarly impacted negatively on reliability and responsiveness. An adequate report on the distribution of scores of the tool, floor and ceiling effects and minimal important change would all enhance the interpretability of scores.

A wealth of methodologies regarding the CCA of HR-PROMs exist. A literature review identified 17 methods of CCA [39] and recently Epstein et al. reported 31 CCA methodologies in a review [8]. Both studies could not establish a gold standard due to the lack of comparative evidence to support one method over another. Recommendations published by Guillemin et al. [6], and refined by Beaton et al. [16], offer a thorough approach to the CCA process and have been applied in systematic reviews of HR-PROMs [10, 14, 40].

A questionable CCA has been observed mainly for the forward translation, back-translation and expert committee review steps. Although apparently counter-intuitive, forward translation and back-translation can receive distinct marks as the CCA steps keep some independence between them when assessed by the methodology selected. There is evidence for methodological inconsistency between studies. For example, while the CCA of the EORTC QLQ-C30 followed the standard EORTC translation procedure (Koller et al. [36], updated by Kulis et al. [41]), the adaptation approach of the EORTC QLQ-C15-PAL (reported by Nunes et al.) differed from the EORTC QoL Group recommendations [25]. The quality of the CCA can be improved through a careful selection of forward translators, a synthesis stage, an expert committee composed of health professionals, translators and possibly patients’ representatives with clear description of their roles, piloting the tool with an ideal sample size (30–40 subjects [16]) and close involvement of developers from the original tool. Future cultural adaptations of PROMs to Brazilian Portuguese should provide sufficient information to permit full quality assessments.

CCV looks for equivalence of scores between source and target population and is an important aspect of PROMs’ performance across cultural groups. Nevertheless, none of the included studies assessed the cross-cultural validity of instruments.

Systematic reviews of clinical trials traditionally stress the methodological appraisal of studies. Likewise, to evaluate the methodological quality of studies assessing HR-PROMs is vital as a well-conducted study on measurement properties leads to reliable results. Different criteria have been proposed to assess the methodological quality of a study’s measurement properties [42–45]. The COSMIN is a consensus-based checklist covering the methodological quality of studies on measurement properties, clearly separating it from the evaluation of the PROM [46]. It provides a scoring system for each measurement property that facilitates direct comparisons between studies [18]. Nevertheless, the checklist does not make clear distinctions between limited methodological quality and poor reporting. Hence, the reasons for poor and fair methodological quality were described in the Results section of this paper for each measurement criterion.

Evidence for positive results on measurement properties with excellent or good methodology was uncommon (17.2%) among studies. The included studies shared similar methodological deficiencies. Methodological aspects that should be improved include the formulation of detailed a priori hypothesis (including the magnitude and direction of expected scores and changes) and good description of comparator instruments, adequate description of missing scores, similar conditions and a reasonable time interval between tests in studies of reliability and measurement error and evaluation of dimensionality in internal consistency analysis.

There is no preferred method to evaluate the performance of HR-PROMs. Terwee et al. have proposed a quality assessment method for measurement properties of HR-PROMs [22], emphasising objective standards that are often lacking in alternative criteria (such as those presented by McDowell and Jenkinson [47]).

None of the PROMs included in this review had all their measurement properties assessed by the papers that evaluated them, and only two had more than half of the measurement properties reported.

The methodological shortcomings observed and the lack of information for some measurement properties limit the applicability of this review. Most importantly, the study results indicate judicious use of any particular tool. Before any definite recommendation or guideline about the “best” tool can be made, the aforementioned improvements in the quality of the CCA and in the methodological aspects of the adapted PROMs should be addressed. Considering the particular qualities and weaknesses of each instrument, our opinion is that the FACT-G version could be recommended for QoL assessment due to the overall excellent methodology (including content validity) and the mostly positive results of its measurement properties. The EORTC QLQ-C30 and QLQ-C15 would be less appropriate to use due to only one positive result among five measurement properties assessed for both instruments (hypothesis testing for EORTC QLQ-C30). For symptom evaluation, the methodological quality and results for measurement properties of MDASI and ESAS were highly variable. Nevertheless, ESAS was more extensively evaluated than MDASI. Moreover, ESAS received two positive results of its measurement properties, while MDASI received negative results for all the properties studied. The VES-13 has a specific population and purpose and should be used accordingly. There is less evidence supporting the use of the SF-36. Poor to fair methodology, negative results in most measurement properties and floor and ceiling effects were observed. Finally, the WHOQOL-bref could not be fully evaluated due to the lack of information about the CCA even after personal contact with the authors.

There are several limitations to this review. The systematic search strategy, albeit thorough, may not have identified all suitable studies. The authors were unable to obtain data regarding the CCA from one of the tools (WHOQOL-bref). The quality analysis of studies reporting on measurement properties has been a topic of continuing debate. Although no gold standard is currently recognised, the methodological quality of the identified studies and the quality of measurement properties were not appraised by any alternative method in this systematic review. The same is true for the CCA analysis. Furthermore, the COSMIN checklist, although widely used, has previously shown limited inter-rater reliability in a validation study [48]. Finally, the potential policy implications of the results were not discussed and could be the focus of future studies.

## Conclusion

This systematic review assessed the availability, CCA quality and measurement properties of Brazilian Portuguese generic PROMs used in the cancer population and cancer-related instruments, aiming at supporting researchers and health practitioners in the selection of questionnaires that suit their particular needs. The critical appraisal of the linguistically and culturally adapted PROMs for use in cancer patients in Brazil indicates limitations to the methodologies used for CCA, and there is limited evidence regarding the quality of their measurement properties. Considering the caveats, we suggest that the adapted FACT-G version could be used for QoL assessments, while the MDASI would be an appropriate tool for symptom evaluation. This review stressed the importance of a systematic approach towards the CCA and the need for consistent adherence to recommended guidelines for robustly developing and adequately testing adapted PROMs.

## Electronic supplementary material

Below is the link to the electronic supplementary material.

Supplementary material 1 (PDF 69 kb)

Supplementary material 2 (PDF 165 kb)
