# Supplementary material for: Cross-cultural adaptation and measurement properties of generic and cancer-related patient-reported outcome measures (PROMs) for use with cancer patients in Brazil: a systematic review
Source: Qual Life Res. 2017 Sep 8;27(4):857–70. doi: 10.1007/s11136-017-1703-5 (PMC5874274; doi:10.1007/s11136-017-1703-5)
Supplement: Supplementary file 1 — Supplementary material 1 (PDF 69 kb) [file 11136_2017_1703_MOESM1_ESM.pdf]

Title: Cross-cultural adaptation and measurement properties of generic and cancer-related patient-reported outcome measures (PROMs) for use with cancer patients in Brazil: a systematic review

Journal name: Quality of Life Research

Authors: Carlos Augusto Albach[a], Richard Wagland, Katherine J Hunt

[a] Corresponding author. MSc candidate, Clinical Leadership in Cancer, Palliative and End of life Care. Faculty of Health Sciences, University of Southampton, University Road, Southampton, Hampshire SO17 1BJ, United Kingdom.

E-mail: caa1n14@soton.ac.uk

Tel: +44(0)23 8059 3131

MEDLINE search strategy (OVID)

1	Questionnaire*.ti,ab.
2	Questionnaire design*.ti,ab.
3	Design, questionnaire*.mp. [mp=title, abstract, original title, name of substance word, subject heading word, protocol supplementary concept, rare disease supplementary concept, unique identifier]
4	Designs, questionnaire.mp. [mp=title, abstract, original title, name of substance word, subject heading word, protocol supplementary concept, rare disease supplementary concept, unique identifier]
5	Instrument*.ti,ab.
6	Scale*.ti,ab.
7	Index*.ti,ab.
8	Score*.ti,ab.
9	Inventory.ti,ab.
10	Evaluat*.ti,ab.
11	Assess*.ti,ab.
12	Survey*.mp. [mp=title, abstract, original title, name of substance word, subject heading word, protocol supplementary concept, rare disease supplementary concept, unique identifier]
13	Measure*.mp. [mp=title, abstract, original title, name of substance word, subject heading word, protocol supplementary concept, rare disease supplementary concept, unique identifier]
14	Tool*.ti,ab.
15	Outcome*.ti,ab.
16	Self-report*.mp. [mp=title, abstract, original title, name of substance word, subject heading word, protocol supplementary concept, rare disease supplementary concept, unique identifier]
17	Report, self.mp. [mp=title, abstract, original title, name of substance word, subject heading word, protocol supplementary concept, rare disease supplementary concept, unique identifier]
18	Reports, self.mp. [mp=title, abstract, original title, name of substance word, subject heading word, protocol supplementary concept, rare disease supplementary concept, unique identifier]
19	Patient-reported.mp. [mp=title, abstract, original title, name of substance word, subject heading word, protocol supplementary concept, rare disease supplementary concept, unique identifier]
20	Report, patient.mp. [mp=title, abstract, original title, name of substance word, subject heading word, protocol supplementary concept, rare disease supplementary concept, unique identifier]

MEDLINE search strategy (OVID)

21	Reports, patient.mp. [mp=title, abstract, original title, name of substance word, subject heading word, protocol supplementary concept, rare disease supplementary concept, unique identifier]
22	Patient centered.ti,ab.
23	Patient centred.ti,ab.
24	Person-reported.ti,ab.
25	Quality of life.mp. [mp=title, abstract, original title, name of substance word, subject heading word, protocol supplementary concept, rare disease supplementary concept, unique identifier]
26	Quality measure*.ti,ab.
27	Quality assessment*.ti,ab.
28	Value of life.mp. [mp=title, abstract, original title, name of substance word, subject heading word, protocol supplementary concept, rare disease supplementary concept, unique identifier]
29	Health status.mp. [mp=title, abstract, original title, name of substance word, subject heading word, protocol supplementary concept, rare disease supplementary concept, unique identifier]
30	Functional status.mp. [mp=title, abstract, original title, name of substance word, subject heading word, protocol supplementary concept, rare disease supplementary concept, unique identifier]
31	QOL.ti,ab.
32	HRQOL.ti,ab.
33	HRQL.ti,ab.
34	PRO.ti,ab.
35	PROM.ti,ab.
36	1 or 2 or 3 or 4 or 5 or 6 or 7 or 8 or 9 or 10 or 11 or 12 or 13 or 14 or 15 or 16 or 17 or 18 or 19 or 20 or 21 or 22 or 23 or 24 or 25 or 26 or 27 or 28 or 29 or 30 or 31 or 32 or 33 or 34 or 35
37	Oncology.mp. [mp=title, abstract, original title, name of substance word, subject heading word, protocol supplementary concept, rare disease supplementary concept, unique identifier]
38	Cancer.mp. [mp=title, abstract, original title, name of substance word, subject heading word, protocol supplementary concept, rare disease supplementary concept, unique identifier]
39	Palliative care.mp. [mp=title, abstract, original title, name of substance word, subject heading word, protocol supplementary concept, rare disease supplementary concept, unique identifier]
40	Palliative.ti,ab.
41	Terminal care.mp. [mp=title, abstract, original title, name of substance word, subject heading word, protocol supplementary concept, rare disease supplementary concept, unique identifier]
42	Terminal*.ti,ab.
43	Tumor*.ti,ab.
44	Neoplasms.mp. [mp=title, abstract, original title, name of substance word, subject heading word, protocol supplementary concept, rare disease supplementary concept, unique identifier]
45	Carcinoma.mp. [mp=title, abstract, original title, name of substance word, subject heading word, protocol supplementary concept, rare disease supplementary concept, unique identifier]
46	Life support*.ti,ab.
47	EOL*.ti,ab.

MEDLINE search strategy (OVID)

48	37 or 38 or 39 or 40 or 41 or 42 or 43 or 44 or 45 or 46 or 47
49	(animals not (humans and animals)).sh.
50	48 not 49
51	36 and 50
52	Valid*.ti,ab.
53	Translat*.ti,ab.
54	Cross-cultural*.ti,ab.
55	Cross-cultural adapt*.ti,ab.
56	Version*.ti,ab.
57	Language.mp. [mp=title, abstract, original title, name of substance word, subject heading word, protocol supplementary concept, rare disease supplementary concept, unique identifier]
58	52 or 53 or 54 or 55 or 56 or 57
59	Brazil*.mp. [mp=title, abstract, original title, name of substance word, subject heading word, protocol supplementary concept, rare disease supplementary concept, unique identifier]
60	Portuguese*.ti,ab.
61	59 or 60
62	51 and 58 and 61

Hits (31st of March 2016): 263
