# Supplementary material for: Cross-cultural adaptation and measurement properties of generic and cancer-related patient-reported outcome measures (PROMs) for use with cancer patients in Brazil: a systematic review
Source: Qual Life Res. 2017 Sep 8;27(4):857–70. doi: 10.1007/s11136-017-1703-5 (PMC5874274; doi:10.1007/s11136-017-1703-5)
Supplement: Supplementary file 2 — Supplementary material 2 (PDF 165 kb) [file 11136_2017_1703_MOESM2_ESM.pdf]

Title: Cross-cultural adaptation and measurement properties of generic and cancer-related patient-reported outcome measures (PROMs) for use with cancer patients in Brazil: a systematic review

Journal name: Quality of Life Research

Authors: Carlos Augusto Albach[a], Richard Wagland, Katherine J Hunt

[a] Corresponding author. MSc candidate, Clinical Leadership in Cancer, Palliative and End of life Care. Faculty of Health Sciences, University of Southampton, University Road, Southampton, Hampshire SO17 1BJ, United Kingdom.

E-mail: caa1n14@soton.ac.uk

Tel: +44(0)23 8059 3131

Excluded studies

Reference	Reason
Albuquerque AS and Tróccoli BT (2004) Development of a Subjective Well-Being Scale. Psicologia: Teoria e Pesquisa 20(2): 153-164	Wrong patient population
Alonso NB, Ciconelli RM, da Silva TI, Westphal-Guitti AC, Azevedo AM, da Silva Noffs MH, Caboclo LOSF, Sakamoto AC and Yacubian EMT (2006) The Portuguese version of the Epilepsy Surgery Inventory (ESI-55): Cross-cultural adaptation and evaluation of psychometric properties. Epilepsy & Behavior 9(1): 126-132	Wrong patient population
Alves E, Medina R and Andreoni C (2013) Validation of the Brazilian version of the Expanded Prostate Cancer Index Composite (EPIC) for patients submitted to radical prostatectomy. International Braz J Urol 39(3): 344-52	Disease or domain-specific PROM
Andrade FP, Antunes JL and Durazzo MD (2006) Evaluation of the quality of life of patients with oral cancer in Brazil. Brazilian Oral Research 20(4): 290-6	Ineligible study design
Araujo J, Dourado M and Ferreira PL (2015) Measuring instruments of the quality of life pediatric palliative care. Acta Medica Portuguesa 28(4): 501-512	Ineligible study design
Ashton-Prolla P, Giacomazzi J, Schmidt AV, Roth FL, Palmero EI, Kalakun L, Aguiar ES, Moreira SM, Batassini E, Belo-Reyes V, Schuler-Faccini L, Giugliani R, Caleffi M and Camey SA (2009) Development and validation of a simple questionnaire for the identification of hereditary breast cancer in primary care. BMC Cancer 9: 283	Tool is not a PROM
Azevedo AM, Alonso NB, Vidal-Dourado M, da Silva Noffs MH, Pascalicchio TF, Caboclo LOSF, Ciconelli RM, Sakamoto AC and Yacubian EMT (2009) Validity and reliability of the Portuguese–Brazilian version of the Quality of Life in Epilepsy Inventory–89. Epilepsy & Behavior 14(3): 465-471	Wrong patient population
Balsanelli ACS, Grossi SAA and Herth KA (2010) Cultural adaptation and validation of the herth hope index for Portuguese language: Study in patients with chronic illness. Texto Contexto Enfermagem 19(4): 754-761	Disease or domain-specific PROM
Baptista RL, Biasoli I, Scheliga A, Soares A, Brabo E, Morais JC, Werneck GL and Spector N (2012) Psychometric properties of the multidimensional fatigue inventory in Brazilian Hodgkin's lymphoma survivors. Journal of Pain & Symptom Management 44(6): 908-15	Wrong patient population
Barros E, Latorre MR, Camargo B, Kurashima AY and Costa CL (2009) Texas revised inventory of grief translation and validation into portuguese: Assessment in bereaved parents who had lost their child due to pediatric cancer. Pediatric Blood and Cancer 53 (5): 867-868	Wrong patient population
Barroso EM, Carvalho AL, Paiva CE, Murphy BA and Paiva BSR (2015) The Vanderbilt Head and Neck Symptom Survey Brazilian Portuguese version 2.0	Disease or domain-specific PROM

Excluded studies

Reference	Reason
(VHNSS 2.0): Psychometric properties for patients with head and neck cancer who have undergone radiotherapy. BMC Res. Notes 8(1)	
Barroso EM, Carvalho AL, Paiva CE, Nunes JS and Paiva BSR (2015) Translation and cross-cultural adaptation into Brazilian Portuguese of the Vanderbilt Head and Neck Symptom Survey version 2.0 (VHNSS 2.0) for the assessment of oral symptoms in head and neck cancer patients submitted to radiotherapy. Brazilian Journal of Otorhinolaryngology 81(6): 622-629	No psychometric property reported
Brabo EP, Paschoal ME, Biasoli I, Nogueira FE, Gomes MC, Gomes IP, Martins LC and Spector N (2006) Brazilian version of the QLQ-LC13 lung cancer module of the European Organization for Research and Treatment of Cancer: preliminary reliability and validity report. Quality of Life Research 15(9): 1519-24	Disease or domain-specific PROM
Busatta SP, Ferreira RA, Klumb-Kiesow LG and Carvalho HW (2014) Preliminary validation study of a Brazilian version of the caregiver reaction assessment in a context of oncologic inpatients. Jornal Brasileiro de Psiquiatria 63(3): 191-199	Wrong patient population
Carvalho AB, Garcia JBS, Silva TKM and Ribeiro JVF (2016) Translation and transcultural adaptation of Pain Quality Assessment Scale (PQAS) to Brazilian version. Revista Brasileira de Anestesiologia 66(1): 94-104	Disease or domain-specific PROM
Carvalho FN, Koifman RJ and Bergmann A (2013) International Classification of Functioning, Disability, and Health in women with breast cancer: a proposal for measurement instruments. Cadernos de Saude Publica 29(6): 1083-93	Tool is not a PROM
Casado L and Thuler LC (2014) Constructing and pretesting a questionnaire to assess the treatment of smokers in Brazilian public health care facilities. Asia-Pacific Journal of Clinical Oncology 10: 93	Tool is not a PROM
Chow E, Nguyen J, Zhang L, Tseng LM, Hou MF, Fairchild A, Vassiliou V, Jesus-Garcia R, Alm El-Din MA, Kumar A, Forges F, Chie WC, Bottomley A and European Organization for Research Treatment of Cancer Quality of Life Group (2012) International field testing of the reliability and validity of the EORTC QLQ-BM22 module to assess health-related quality of life in patients with bone metastases. Cancer 118(5): 1457-65	Wrong patient population
Ciconelli RM, Soárez PC, Kowalski CCG and Ferraz MB (2006) The Brazilian Portuguese version of the Work Productivity and Activity Impairment: General Health (WPAI-GH) Questionnaire. Sao Paulo Medical Journal 124(6): 325-332	Wrong patient population
Dias RS, Ramos CC, Correa FK, Trinca LA, Cerqueira ATAR, Dalben I and Moreno RA (2002) The Brazilian Portuguese criterion validation of the Women's Health Questionnaire (WHQ) - Mid-aged women's perception of their emotional and physical health. Revista de Psiquiatria Clinica 29(4): 181-189	Wrong patient population
da Silva FC, Thuler LCS and Leon-Casasola OA (2011) Validity and reliability of two pain assessment tools in Brazilian children and adolescents. Journal of Clinical Nursing 20(13-14): 1842-8	Tool is not a PROM
Simao DAS, Teixeira AL, Souza RS and Lima EDP (2014) Evaluation of the Semmes-Weinstein filaments and a questionnaire to assess chemotherapy-induced peripheral neuropathy. Supportive Care in Cancer 22(10): 2767-73	Disease or domain-specific PROM
Damásio BF, Borsa JC and Koller SH (2014) Adaptation and psychometric properties of the Brazilian Version of the Five-item Mental Health Index (MHI-5). Psicologia: Reflexão e Crítica 27(2): 323-330	Wrong patient population
Damásio BF, Pacico JC, Poletto M and Koller SH (2013) Refinement and psychometric properties of the eight-item Brazilian Positive and Negative Affective Schedule for Children (PANAS-C8). Journal of Happiness Studies 14(4): 1363-1378	Wrong patient population

Excluded studies

Reference	Reason
de Andrade FP, Biazevic MG, Toporcov TN, Togni J, de Carvalho MB and Antunes JL (2012) Discriminant validity of the University of Washington quality of life questionnaire in the Brazilian context. Revista Brasileira de Epidemiologia 15(4): 781-9	Disease or domain-specific PROM
De Carlo MMRP and Correia FR (2014) Preliminary validation of the brazilian version of the palliative care outcome scale (POS-Br). Palliative Medicine 28 (6): 823-824	Conference abstract
de Carvalho HW, Andreoli SB, Lara DR, Patrick CJ, Quintana MI, Bressan RA, de Melo MF, Mari JJ and Jorge MR (2013) Structural validity and reliability of the Positive and Negative Affect Schedule (PANAS): Evidence from a large Brazilian community sample. Revista Brasileira de Psiquiatria 35(2): 169-172	Wrong patient population
De Lima RAG, Nunes MDR, Silva MCM, Rocha EL and Nascimento LC (2012) Validation of the PEDSQL Multidimensional Fatigue Scale in Brazilian children and adolescents with cancer: Initial psychometric properties. Supportive Care in Cancer 20: S126	Wrong patient population
De Souza CA, Vigorito AC, Ruiz MA, Nucci M, Dulley FL, Funcke V, Tabak D, Azevedo AM, Byington R, Macedo MC, Saboya R, Aranha FJP, Oliveira GB, Zulli R, Miranda ECM, Azevedo WM, Lodi FM, Voltarelli JC, Simoes BP, Colturato V, De Souza MP, Silla L, Bittencourt H, Piron-Ruiz L, Maiolino A, Gratwohl A and Pasquini R (2005) Validation of the EBMT risk score in chronic myeloid leukemia in Brazil and allogeneic transplant outcome. Haematologica 90(2): 232-237	Tool is not a PROM
de Souza RF, Leles CR, Guyatt GH, Pontes CB, Della Vecchia MP and Neves ND (2010) Exploratory factor analysis of the Brazilian OHIP for edentulous subjects. Journal of Oral Rehabilitation 37(3): 202-208	Wrong patient population
Decat CSA, Laros JA and Araujo TCCF (2009) Termômetro de Distress: validação de um instrumento breve para avaliação diagnóstica de pacientes oncológicos. Psico-USF 14(3): 253-260	Ineligible study design
Decat CSA, Laros JA, Araujo TCCF, Buso MM and Nonino A (2009) Distress thermometer: Adaptation and validation study of a brief screening instrument to detect distress in cancer patients. Psycho-Oncology 18: S319	Conference abstract
Delalibera M, Coelho A and Barbosa A (2011) Validation of prolonged grief disorder instrument for Portuguese population. Acta Medica Portuguesa 24(6): 935-42	Wrong patient population
Dettino AA, Pontes LL, Pagano T, Fanelli MF, Chinen LT and Costa CL (2011) Distress in gastrointestinal cancer: Distress thermometer (DT, NCCN)-Validation to Brazilian Portuguese. Journal of Clinical Oncology. Conference 29(4 SUPPL. 1)	Conference abstract
Dini EL, McGrath C and Bedi R (2003) An evaluation of the Oral Health Quality of Life (OHQoL) instrument in a Brazilian population. Community Dental Health 20(1): 40-44	Full text unavailable/Author unreachable
Dos Santos J, Kurita GP and Pimenta CAM (2011) A systematic review on cognitive assessment of patients with cancer in palliative care. Supportive Care in Cancer (1): S135	Tool is not a PROM
Dos Santos J, Kurita GP, Pimenta CAP, Braga PE, Lundorff L, Ekholm O and Sjogren P (2012) Neuropsychological measurement in cancer patients: A validation study. Palliative Medicine 26 (4): 473-474	Tool is not a PROM
Campos JADB and do Prado CD (2012) Cross-cultural adaptation of the Portuguese version of the Patient-Generated Subjective Global Assessment. Nutricion Hospitalaria 27(2): 583-9	Tool is not a PROM

Excluded studies

Reference	Reason
Duarte PS, Ciconelli RM and Sesso R (2005) Cultural adaptation and validation of the 'Kidney Disease and Quality of Life - Short Form (KDQOL-SFTM 1.3)' in Brazil. Brazilian Journal of Medical and Biological Research 38(2): 261-270	Disease or domain-specific PROM
Duarte PS, Miyazaki MCOS, Ciconelli RM and Sesso R (2003) Tradução e adaptação cultural do instrumento de avaliação de qualidade de vida para pacientes renais crônicos (KDQOL-SF TM). Revista da Associação Médica Brasileira 49(4): 375-381	No psychometric property reported
Ferreira KA, Teixeira MJ, Mendonza TR and Cleeland CS (2011) Validation of brief pain inventory to Brazilian patients with pain. Supportive Care in Cancer 19(4): 505-11	Disease or domain-specific PROM
Ferreira KASL, Júnior WNW, Mendonza TR, Kimura M, Kowalski LP, Rosenthal DI and Cleeland CS (2008) Translation of the M.D. Anderson Symptom Inventory - head and neck module (MDASI-HN) to Brazilian Portuguese. Rev. bras. cir. cabeça pescoço 37(2): 109-113	No psychometric property reported
Ferreira PL and Anes EJ (2010) Medição da qualidade de vida de insuficientes renais crónicos: criação da versão portuguesa do KDQOL-SF. Revista Portuguesa de Saúde Pública 28(1): 31-39	Wrong patient population
Franceschini J, Jardim JR, Fernandes AL, Jamnik S and Santoro IL (2010) Reproducibility of the Brazilian Portuguese version of the European Organization for Research and Treatment of Cancer Core Quality of Life Questionnaire used in conjunction with its lung cancer-specific module. Jornal Brasileiro De Pneumologia 36(5): 595-602	Disease or domain-specific PROM
Fregnani CM, Fregnani JH, Latorre MRDO and de Almeida AM (2013) Evaluation of the psychometric properties of the Functional Assessment of Cancer Therapy-Cervix questionnaire in Brazil. PLoS ONE [Electronic Resource] 8(10): e77947	Disease or domain-specific PROM
Fumis RR, Nishimoto IN and Deheinzelin D (2006) Measuring satisfaction in family members of critically ill cancer patients in Brazil. Intensive Care Medicine 32(1): 124-8	Wrong patient population
Gazzotti MR, Alith MB, Malheiros SM, Vidotto MC, Jardim JR and Nascimento OA (2011) Functional assessment of cancer therapy-brain questionnaire: translation and linguistic adaptation to Brazilian Portuguese. Sao Paulo Medical Journal 129(4): 230-5	Disease or domain-specific PROM
Geronutti D, Murra M, Lopes LF and Paiva BS (2014) Identification of instruments used to evaluate symptoms in children and adolescents with cancer-systematic review. Pediatric Blood and Cancer 61: S347	Conference abstract
Gonçalves CO, Tavares MCGCF, Campana ANNB and Cabello C (2014) Validation of the instrument "Body image after breast cancer" in Brazil. Motriz: Revista de Educação Física 20(1): 8-15	Disease or domain-specific PROM
Goncalves DM and Cloninger CR (2010) Validation and normative studies of the Brazilian Portuguese and American versions of the Temperament and Character Inventory—Revised (TCI-R). Journal of Affective Disorders 124(1-2): 126-133	Wrong patient population
Gouveia VV, Milfont TL, da Fonseca PN and Coelho JAPM (2009) Life satisfaction in Brazil: Testing the psychometric properties of the Satisfaction with Life Scale (SWLS) in five Brazilian samples. Social Indicators Research 90(2): 267-277	Wrong patient population
Guedes RL, Angelis EC, Chen AY, Kowalski LP and Vartanian JG (2013) Validation and application of the M.D. Anderson Dysphagia Inventory in patients treated for head and neck cancer in Brazil. Dysphagia 28(1): 24-32	Disease or domain-specific PROM
Heldwein FL, Traebert JL, Hartmann AA, Pioner GT and Teloken C (2015) RE: Validation of the brazilian version of the expanded prostate cancer index composite	Letter to the editor

Excluded studies

Reference	Reason
(EPIC) for patients submitted to radical prostatectomy. International Brazilian Journal of Urology: 41(3): 604-605	
Imada TCML, Mamede MV, Souza L and Biffi RG (2010) Adaptação e validação da Family Dynamics Measure II para familiares de mulheres com câncer de mama. Psicologia: Teoria e Pesquisa 26(3): 557-564	Tool is not a PROM
Ishikawa NM, Thuler LC, Giglio AG, Baldotto CS, de Andrade CJ and Derchain SF (2010) Validation of the Portuguese version of functional assessment of cancer therapy-fatigue (FACT-F) in Brazilian cancer patients. Supportive Care in Cancer 18(4): 481-90	Disease or domain-specific PROM
Juliana F, Jardim JR, Fernandes AL, Jamnik S and Santoro IL (2010) Reliability of the Brazilian version of the Functional Assessment of Cancer Therapy-Lung (FACT-L) and the FACT-Lung Symptom Index (FLSI). Clinics 65(12): 1247-51	Disease or domain-specific PROM
Kurashima AY, Latorre MR and Camargo B (2010) A palliative prognostic score for terminally ill children and adolescents with cancer. Pediatric Blood & Cancer 55(6): 1167-71	Tool is not a PROM
Lessa PRA (2012) Translation, adaptation and validation of the scale Adherence Determinants Questionnaire for use in Brazil [manuscript]. Unpublished MSc thesis Universidade Federal do Ceará University	Thesis
Lessa PRA, Ribeiro SG, Aquino PS, de Almeida PC and Pinheiro AKB (2015) Validation of the Adherence Determinants Questionnaire scale among women with breast and cervical cancer. Revista Latino-Americana de Enfermagem 23(5): 971-978	Disease or domain-specific PROM
Lima AS, Barros L and Enumo SRF (2014) Coping in Portuguese children hospitalized for cancer: a comparison of two assessment instruments. Estudos Psicológicos 31(4): 559-571	Wrong patient population
Lima EDRP, Norman EM and Lima AP (2005) Translation and adaptation of the Social Support Network Inventory in Brazil. Journal of Nursing Scholarship 37(3): 258-60	Disease or domain-specific PROM
Lopes M, Koch VHK and Varni JW (2011) Tradução e adaptação cultural do Peds QL TM ESRD para a língua portuguesa<br>Translation and cultural adaptation of Peds QL TM ESRD to Portuguese. Jornal Brasileiro de Nefrologia 33(4): 448-456	No psychometric property reported
Luz LL (2015) Multidimensional assessment of health of elderly patients with prostate cancer and The Vulnerable Elders Survey 13 (VES-13) as a screening tool in oncogeriatria. [manuscript] Unpublished MSc thesis Universidade Federal do Rio de Janeiro University	Thesis
Luz LL, Santiago LM, Silva JF and Mattos IE (2013) First stage of the cross-cultural adaptation of the instrument The Vulnerable Elders Survey (VES-13) to Portuguese. Cadernos de Saude Publica 29(3): 621-8	No psychometric property reported
Martinez MC, Latorre MRDO and Fischer FM (2009) Validity and reliability of the Brazilian version of the Work Ability Index questionnaire. Revista de Saude Publica 43(3): 525-532	Wrong patient population
Mastropietro AP, Oliveira EA, Santos MA and Voltarelli JC (2007) Functional Assessment of Cancer Therapy Bone Marrow Transplantation: Portuguese translation and validation. Revista de Saude Publica 41(2): 260-8	Disease or domain-specific PROM
Mathias C, Athanazio RA, Braghiroli MI, Nunez G, Lessa R, Macedo G, De Sena EP and Del Giglio A (2005) Use of Arizona Sexual Experience Scale (ASEX) for the evaluation of sexual dysfunction in Brazilian oncologic patients. Jornal Brasileiro de Psiquiatria 54(3): 216-220	Disease or domain-specific PROM

Excluded studies

Reference	Reason
Matias KS, Lehn CN and Antunes JLF (2007) Quality of life of patients with oral and oropharynx cancer using the questionnarie UW-QOL. Revista de Pós Graduação 14(2): 139-146	Ineligible study design
Mayoral VF, Fukushima FB, Jacinto AF, Villas-Boas PJ, Carvalho R, Rodrigues A, Carvalho L, Polegato B, Minicucci M, Pinheiro L and Vidal EI (2015) Cross-cultural adaptation of the POLST form to Brazil. Journal of the American Geriatrics Society 63: S253-S254	Tool is not a PROM
Mesquita AC, Simao TP, Chaves ECL, Carvalho CC, Carvalho EC, Ku YL and Iunes DH (2013) Cultural adaptation and validation of spiritual distress scale. European Journal of Cancer 49: S390	Conference abstract
Michels FA, Latorre MR and Maciel MS (2013) Validity, reliability and understanding of the EORTC-C30 and EORTC-BR23, quality of life questionnaires specific for breast cancer. Revista Brasileira de Epidemiologia 16(2): 352-63	Disease or domain-specific PROM
Michels FA, Latorre MDRDDO and Maciel MDS (2012) Validity and reliability of the FACT-B+4 quality of life questionnaire specific for breast cancer and comparison of IBCSG, EORTC-BR23 and FACT-B+4 questionnaires. Cadernos de Saude Coletiva 20(3): 321-328	Disease or domain-specific PROM
Monteiro DR, Almeida MA and Kruse MH (2013) Translation and cross-cultural adaptation of the Edmonton Symptom Assessment System for use in Palliative Care. Revista gaucha de enfermagem 34(2): 163-171	No psychometric property reported
Monteiro DR, Kruse MH and Almeida MA (2010) Assessment of the instrument Edmonton Symptom Assessment System in hospice care: an integrative review. Revista gaucha de enfermagem 31(4): 785-793	Ineligible study design
Monteiro IS and Maia AC (2010) Avaliação psicométrica de três questionários sobre o historial familiar. Archives of Clinical Psychiatry 37(3): 97-104	Tool is not a PROM
Moreira CA, Junior WG, Lima LF, Lima CR, Ribeiro JF and Miranda AF (2009) Assesment or the basic psychometric properties for the portuguese version of the KDQOL-SF. Revista da Associação Médica Brasileira 55(1): 22-28	Disease or domain-specific PROM
Morete MC, Mofatto SC, Pereira CA, Silva AP and Odierna MT (2014) Translation and cultural adaptation of the Brazilian Portuguese version of the Behavioral Pain Scale. Revista Brasileira de Terapia Intensiva 26(4): 373-378	Wrong patient population
Mota DD, Pimenta CA and Piper BF (2009) Fatigue in Brazilian cancer patients, caregivers, and nursing students: a psychometric validation study of the Piper Fatigue Scale-Revised. Supportive Care in Cancer 17(6): 645-52	Disease or domain-specific PROM
Mota DDCF, Pimenta CAM and Fitch MI (2009) Pictograma de Fadiga: uma alternativa para avaliação da intensidade e impacto da fadiga. Revista da Escola de Enfermagem da USP 43: 1080-1087	Disease or domain-specific PROM
Nascimento LC, Nunes MDR, Rocha EL, Bomfim EO, Floria-Santos M, dos Santos CB, de Souza Serio dos Santos DM and de Lima RAG (2015) High validity and reliability of the PedsQ™ Multidimensional Fatigue Scale for Brazilian children with cancer. Journal of Pediatric Oncology Nursing 32(1): 57-64	Wrong patient population
Nascimento MI, Reichenheim ME and Monteiro GT (2011) Dimensional structure of the Brazilian version of the Scale of Satisfaction with Interpersonal Processes of General Medical Care. Cadernos de Saude Publica 27(12): 2351-63	Wrong patient population
Nassar Junior AP, Pires Neto RC, Figueiredo WB and Park M Validity, reliability and applicability of Portuguese versions of sedation-agitation scales among critically ill patients. Sao Paulo Medical Journal 126(4): 215-219	Tool is not a PROM
Naylor C, Cerqueira L, Costa-Paiva LH, Costa JV, Conde DM and Pinto-Neto AM (2010) Survival of women with cancer in palliative care: use of the palliative	Tool is not a PROM

Excluded studies

Reference	Reason
prognostic score in a population of Brazilian women. Journal of Pain & Symptom Management 39(1): 69-75	
Nunes MDR, Silva MCM, Rocha EL, de Lima RAG and Nascimento LC (2014) Measurement of fatigue in children and adolescents with cancer: An integrative review. Texto Contexto Enfermagem 23(2): 492-501	Ineligible study design
O'Connell KA, Saxena S and Skevington SM (2004) WHOQOL-HIV for quality of life assessment among people living with HIV and AIDS: Results from the field test. AIDS Care 16(7): 882-889	Disease or domain-specific PROM
Oliveira MZ, Zanon C, Silva IS, Pinhatti MM, Gomes WB and Gauer G (2003) Validation of the brazilian version of the Boundaryless Career Attitudes Scale. Arquivos Brasileiros de Psicologia 62(3): 106-114	Wrong patient population
Osorio FL, Lima MP and Chagas MH (2015) Assessment and screening of panic disorder in cancer patients: performance of the PHQ-PD. Journal of Psychosomatic Research 78(1): 91-4	Disease or domain-specific PROM
Paiva BS, de Carvalho AL, Kolcaba K and Paiva CE (2015) Validation of the Holistic Comfort Questionnaire-caregiver in Portuguese-Brazil in a cohort of informal caregivers of palliative care cancer patients. Supportive Care in Cancer 23(2): 343-51	Wrong patient population
Paiva CE, Rugno FC and Paiva BS (2012) The Barretos short instrument for assessment of quality of life (BSIqol): development and preliminary validation in a cohort of cancer patients undergoing antineoplastic treatment. Health & Quality of Life Outcomes 10(144): 1-8	New tool developed in Brazil
Paiva CE, Siquelli FAF, Santos HA, Costa MM, Massaro DR, Lacerda DC, Nunes JS, de Padua Souza C and Paiva BSR (2015) The Functionality Assessment Flowchart (FAF): A new simple and reliable method to measure performance status with a high percentage of agreement between observers. BMC Cancer 15 (1) (no pagination)	New tool developed in Brazil
Pan R, Marques AR, Santos BD, Jacob E, Santos CB and Nascimento LC (2014) Cultural adaptation to Brazil of the questionnaire Costs of caring for children with cancer. Revista Latino-Americana de Enfermagem 22(4): 591-597	Tool is not a PROM
Pasin S, Avila F, De Cavata T, Hunt A and Heldt E (2013) Cross-cultural translation and adaptation to Brazilian Portuguese of the paediatric pain profile in children with severe cerebral palsy. Journal of Pain and Symptom Management 45(1): 120-128	Tool is not a PROM
Pastore CA, Orlandi SP and Gonzalez MC (2014) The Inflammatory-Nutritional Index: assessing nutritional status and prognosis in gastrointestinal and lung cancer patients. Nutricion Hospitalaria 29(3): 629-34	Tool is not a PROM
Pereira FM and Santos CS (2014) Initial validation of the Mini-Mental Adjustment to Cancer (Mini-MAC) scale: study of Portuguese end-of-life cancer patients. European journal of oncology nursing 18(5): 534-539	Wrong patient population
Pimenta CA and da Cruz DA (2006) Chronic pain beliefs: validation of the survey of pain attitudes for the Portuguese language. Revista Da Escola de Enfermagem Da USP 40(3): 365-73	Wrong patient population
Pontes RMA Quality of life with inflammatory bowel diseases: translation to portuguese language and validation of the Inflammatory Bowel Disease Questionnaire (IBDQ). [manuscript] 1999	Thesis
Rebolledo DC, Vissoci JR, Pietrobon R, de Camargo OP and Baptista AM (2013) Validation of the Brazilian version of the musculoskeletal tumor society rating scale for lower extremity bone sarcoma. Clinical Orthopaedics & Related Research 471(12): 4020-6	Wrong patient population

Excluded studies

Reference	Reason
Rhoden EL, Teloken C, Sogari PR and Souto CAV (2002) The use of the simplified International Index of Erectile Function (IIEF-5) as a diagnostic tool to study the prevalence of erectile dysfunction. International Journal of Impotence Research 14(4): 245-50	Tool is not a PROM
Santos CB, Carvalho SC, Silva MF, Fuentes D, Santana PA, Furlan AB and Aguiar PH (2008) Cross-cultural adaptation of the Innsbruck Health Dimensions Questionnaire for Neurosurgical Patients (IHD-NS). Arquivos de Neuro-Psiquiatria 66(3B): 698-701	Disease or domain-specific PROM
Santos HHANM, Aguiar AGO, Baeck HE and Van Borsel J Translation and preliminary evaluation of the Brazilian Portuguese version of the Transgender Voice Questionnaire for male-to-female transsexuals. CoDAS 27(1): 89-96	Wrong patient population
Santos J, Kurita GP, Pimenta CAM, Braga PE, Lundorff L, Ekholm O and Sjogren P (2012) Neuropsychological measurement in cancer patients: Validation of trail making test. Supportive Care in Cancer 20: S54	Tool is not a PROM
Saraiva D, de Camargo B and Davis AM (2008) Cultural adaptation, translation and validation of a functional outcome questionnaire (TESS) to Portuguese with application to patients with lower extremity osteosarcoma. Pediatric Blood & Cancer 50(5): 1039-42	Wrong patient population
Saraiva DCA, Afonso WV, Pinho NB, Peres WAF and Padilha PC (2016) Equivalência semântica do Questionário Pediatric Subjective Global Nutritional Assessment para triagem nutricional em pacientes pediátricos com câncer. Revista de Nutrição 29(2): 211-227	Tool is not a PROM
Sartore AC and Grossi SA (2008) Herth Hope Index instrument adapted and validated to Portuguese. Revista Da Escola de Enfermagem Da USP 42(2): 227-32	Wrong patient population
Scagliusi FB, Polacow VO, Cordas TA, Coelho D, Alvarenga M, Philippi ST and Lancha Jr AH (2006) Translation, adaptation and psychometric evaluation of the National Health Interview Survey Cancer Epidemiology Nutrition Knowledge Scale. Revista de Nutricao 19(4): 425-436	Tool is not a PROM
Scarpelli AC, Paiva SM, Pordeus IA, Ramos-Jorge ML, Varni JW and Allison PJ (2008) Measurement properties of the Brazilian version of the Pediatric Quality of Life Inventory (PedsQL) cancer module scale. Health & Quality of Life Outcomes 6: 7	Wrong patient population
Scarpelli AC, Paiva SM, Pordeus IA, Varni JW, Viegas CM and Allison PJ (2008) The pediatric quality of life inventory (PedsQL) family impact module: reliability and validity of the Brazilian version. Health & Quality of Life Outcomes 6: 35	Wrong patient population
Shimoda S, de Camargo B, Horsman J, Furlong W, Lopes LF, Seber A and Barr RD (2005) Translation and cultural adaptation of Health Utilities Index (HUI) Mark 2 (HUI2) and Mark 3 (HUI3) with application to survivors of childhood cancer in Brazil. Quality of Life Research 14(5): 1407-12	Wrong patient population
Silva FC and Thuler LC (2008) Cross-cultural adaptation and translation of two pain assessment tools in children and adolescents. Jornal de Pediatria 84(4): 344-9	Tool is not a PROM
Silva LF (2015) Validated questionnaires to assess sexual function of survivors of the uterine cervical neoplasm. Journal of Sexual Medicine 12: 95-96	Ineligible study design
Silva TI, Marques CM, Alonso NB, Azevedo AM, Westphal-Guitti AC, Caboclo LOSF, Sakamoto AC and Yacubian EMT (2006) Tradução e adaptação cultural do Quality of Life in Epilepsy (QOLIE-31). Journal of Epilepsy and Clinical Neurophysiology 12(2): 107-110	Wrong patient population
Silveira A, Goncalves J, Sequeira T, Ribeiro C, Lopes C, Monteiro E and Pimentel FL (2011) Computer-based quality-of-life monitoring in head and neck cancer	Wrong patient population

Excluded studies

Reference	Reason
patients: A validation model using the EORTC-QLQ C30 and EORTC- H&N35 Portuguese PC-software version. Acta Medica Portuguesa 24(SUPPL.2): 347-354	
Simão TP, Chaves ECL, Carvalho EC, Nogueira DA, Carvalho CC, Ku YL and Iunes DH (2016) Cultural adaptation and analysis of the psychometric properties of the Brazilian version of the Spiritual Distress Scale. Journal of Clinical Nursing 25(1-2): 231-239	Disease or domain-specific PROM
Soares A, Biasoli I, Scheliga A, Baptista RL, Brabo EP, Morais JC, Werneck GL and Spector N (2012) Validation of the Brazilian portuguese version of the medical outcomes study-social support survey in Hodgkin's lymphoma survivors. Supportive Care in Cancer 20(8): 1895-1900	Wrong patient population
Soares M, Fontes F, Dantas J, Gadelha D, Cariello P, Nardes F, Amorim C, Toscano L and Rocco JR (2004) Performance of six severity-of-illness scores in cancer patients requiring admission to the intensive care unit: a prospective observational study. Critical Care 8(4): R194-203	Tool is not a PROM
Soares M, Silva UV, Teles JM, Silva E, Caruso P, Lobo SM, Dal Pizzol F, Azevedo LP, de Carvalho FB and Salluh JI (2010) Validation of four prognostic scores in patients with cancer admitted to Brazilian intensive care units: results from a prospective multicenter study. Intensive Care Medicine 36(7): 1188-95	Tool is not a PROM
Spanemberg L, Salum GA, Caldieraro MA, Vares EA, Tiecher RD, da Rocha NS, Parker G and Fleck MP (2014) Personality styles in depression: Testing reliability and validity of hierarchically organized constructs. Personality and Individual Differences 70: 72-79	Wrong patient population
Spexoto MCB, Serrano SV, Maroco J and Campos JADB (2013) Quality of life of cancer patients: Face validity and content validity of two instruments. Psychotherapy and Psychosomatics 82: 107-108	Conference abstract
Spexoto MCB, Serrano SV, Halliday V, Maroco J and Campos JADB (2016) Cancer Appetite and Symptom Questionnaire (CASQ) for Brazilian patients: Cross-cultural adaptation and validation study. PLoS ONE 11(6)	Disease or domain-specific PROM
Strazzieri-Pulido KC, Santos VLCG and Carville K (2015) Cultural adaptation, content validity and inter-rater reliability of the STAR Skin Tear Classification System. Revista Latino-Americana de Enfermagem 23(1): 155-161	Tool is not a PROM
Toledo FO, Barros PS, Herdman M, Vilagut G, Reis GC, Alonso J and Ferreira Da Rosa Sobreira C (2013) Cross-cultural adaptation and validation of the Brazilian version of the Wisconsin brief pain questionnaire. Journal of Pain and Symptom Management 46(1): 121-130	Wrong patient population
Trotte LAC, Lima CFM, Pena TLN, Ferreira AMO and Caldas CP (2014) Cross-cultural adaptation of the End of Life Comfort Questionnaire-Patient to Brazilian Portuguese. Revista de Enfermagem 22(4): 461-465	Disease or domain-specific PROM
Vartanian JG, Carvalho AL, Yueh B, Furia CL, Toyota J, McDowell JA, Weymuller EA, Jr. and Kowalski LP (2006) Brazilian-Portuguese validation of the University of Washington Quality of Life Questionnaire for patients with head and neck cancer. Head & Neck 28(12): 1115-21	Disease or domain-specific PROM
Vieira EM, Dos Santos MA, Santos DB, Mancini MPM, Souza HCC, Bazan JL and Perdoná GSC (2015) Validation of body image relationship scale for women with breast cancer. Revista Brasileira de Ginecologia e Obstetricia 37(10): 473-479	Disease or domain-specific PROM
